# Additional evidence from a case report supports a novel hypothesis on the association between complex regional pain syndrome and lymphedema

**DOI:** 10.3389/fpain.2025.1540930

**Published:** 2025-06-26

**Authors:** M. Mark Melin, John C. Rasmussen, Melissa B. Aldrich, Ron J. Karni, Caroline E. Fife, Kristen A. Eckert

**Affiliations:** ^1^ Gonda Vascular Center, Wound Clinic, Mayo Clinic, Rochester, MN, United States; ^2^ Brown Foundation Institute of Molecular Medicine, McGovern Medical School, The University of Texas Health Science Center at Houston, Houston, TX, United States; ^3^ Division of Head and Neck Surgical Oncology, Department of Otorhinolaryngology, McGovern Medical School, The University of Texas Health Science Center at Houston, Houston, TX, United States; ^4^ Intellicure, Inc., The Woodlands, TX, United States; ^5^ Department of Internal Medicine, Division of Geriatrics, Baylor College of Medicine, Houston, TX, United States; ^6^ Strategic Solutions, Inc., Bozeman, MT, United States

**Keywords:** manual lymphatic drainage, automated MLD therapy, complex regional pain syndrome, autonomic dysfunction, lymphatic dysfunction, lymphatic contractility, pain, near-infrared lymphatic imaging

## Abstract

A previous report of 4 heterogeneous cases demonstrated that automated manual lymphatic drainage therapy (AMLDT), administered by a novel, pneumatic mat of 16 pressurized air channels that inflate and deflate sequentially to mimic the stretch and release action of manual lymphatic drainage therapy (MLD), altered lymphatic contractility and relieved pain. Near-infrared fluorescence imaging (NIRF-LI) was used 1 h before AMLDT, during 1 h of AMDLT, and 30–60 min after treatment to obtain images that could be used to determine lymphatic contractility, as measured by pulsing frequency over a given timeframe. Herein, a case of type 2 complex regional pain syndrome (CRPS, with nerve dysfunction confirmed) and lymphedema following a complex fracture on the lower leg is reported in further detail, with a discussion explaining the association between autonomic and lymphatic dysfunction and their combined contribution to the development of chronic pain. More specifically, this case provides clinical evidence of the association between autonomic nervous system dysfunction, lymphatic dysfunction, and CRPS. We believe that the regulation of lymphatic flow is a potential therapeutic pathway to alleviate the symptoms of CRPS. Further research on the association between autonomic and lymphatic dysfunction and pain is warranted, particularly in patients with CRPS and symptoms of edema following leg fractures.

## 1 Introduction

Every year, approximately 29 per 100,000 people develop complex regional pain syndrome (CRPS), a systemic disorder involving chronic pain and inflammation usually triggered by a traumatic injury of the extremities and manifesting as edema, altered dermal temperature and color, digit and/or limb dystrophy, and allodynia (1–5). Fractures, crush injuries, sprains, and surgery are the most frequent reasons for CRPS development (1, 6–8). There are 2 types of CRPS: type 1 occurs without a triggering nerve injury, while type 2 develops after a nerve injury. Treatment options are very limited and do not provide long-term pain relief. The United States Food and Drug Administration has not approved a pharmacological treatment for this debilitating condition, although multiple pharmacologic treatments have been recommended, including nonsteroidal anti-inflammatory drugs, steroids, gabapentin, bisphosphonates, ketamine, botulinum toxin A, and various antioxidants (9, 10).

A complex and highly integrated cascading chain of inflammatory and neuroimmune responses are thought to contribute to CRPS development, with an initial proinflammatory cytokine storm observed following injury (1, 11–14). Acute CRPS is characterized by reduced levels of norepinephrine, a stress transmitter of the sympathetic nervous system (SNS) and increased levels of α-1 adrenergic receptors. These in turn cause increased blood flow and vasodilation in the affected extremity (7, 15–17), resulting in the production of more proinflammatory interleukin-16 (IL-16). Elevated IL-16 induces B lymphocytes and increases antibody production, including immunoglobulin G and M (IgG and IgM) (13, 18, 19). As CRPS progresses to a chronic condition, the persistent proliferation of proinflammatory cytokines further increases norepinephrine levels, reducing α-1 adrenergic receptors, and resulting in a persistent vasoconstriction. Additionally, there is a heightened immune response that is characterized by dysfunctional T-cell activity and exaggerated neuroinflammation and nociception, mediated by altered IgG and IgM antibodies (1, 11, 12).

The hallmark symptom of CRPS is a regional, nociplastic pain that is disproportionate to the initial injury, without damaged tissue or dysfunctional somatosensory system, and it can manifest in contralateral “uninjured” extremities (1, 20, 5, 21–53). Nociplastic pain is a type of pain in which augmented central nervous system pain and sensory processing and altered pain modulation play prominent roles (24). Although nociplastic pain is most associated with CRPS at the time of diagnosis, affected patients can also feel nociceptive pain (caused by the elevated nociceptors in nonneural tissue) and neuropathic pain, as well as mixed pain (21).

Researchers increasingly understand that the autonomic nervous system (ANS) and the lymphatic system work together in a highly coordinated, cellular-molecular response to immunogenic threats and support vascular and lymphatic homeostasis via neurotransmitter and neuropeptide communications within the lymph nodes (25). *In vivo* studies provide evidence of the association between ANS neurotransmitters and lymphatic dysfunction. When norepinephrine was injected into mice, SNS nerve fibers in the lymph node capsule and parenchyma drastically increased lymph flow, demonstrating crosstalk occurs between the SNS and the lymphatic system (26). Sympathectomy treatment has been found to block the activated SNS in the lymph nodes, reducing lymph flow by as much as 80% (26–28). Murine models have also demonstrated that muscarinic and α-adrenergic agonists promote lymphatic contractility, while β_2_-adrenergic agonists decrease contractility (27).

In a murine limb fracture model, popliteal lymph nodes from the affected extremity had IgM deposits, which were resolved following chemical sympathectomy (29). Consequently, CRPS has been referred to as localized autoimmune syndrome mediated by autoantibodies and involving ANS dysfunction (1, 6, 7, 15–17). Therefore, the current hypothesis is that both autonomic and lymphatic function contribute to CRPS (14), which further suggests that the regulation of lymph flow is a potential therapeutic pathway for CRPS.

In alignment with the evolution of CRPS understanding as a manifestation of an autoimmune disease, *in vivo* studies have demonstrated the CRPS is associated with lymphatic dysfunction (29, 31–33). When the lymphatic system fails to achieve fluid homeostasis in the body, there is a buildup of interstitial fluid containing noxious inflammatory cytokines and corresponding increased microvascular permeability leakage (33). The cytokines persistently activate nociceptors and interfere with normal lymphatic responsiveness and pumping following tissue injury, rapidly decreasing lymphatic flow and pulsatile frequency, leading to stasis and a chronic feed-forward loop of musculoskeletal and/or nonmusculoskeletal nociceptors hypothesized to be responsible for chronic pain (14, 33). The accumulated proinflammatory cytokines in the interstitial fluid, IL-1β, IL-6, and tumor necrosis factor-α, are in fact the same ones responsible for feeling pain (14, 35, 36). Over time, this chronic and painful inflammatory response manifests as lymphedema (LE) (14, 31–33). LE is prevalent in more than half of patients who have had leg fractures, 14% of whom have recurrent infections indicative of severe LE (37). Additionally, this patient population is at risk of developing CRPS (1, 6–8). Lymphoscintigraphy has confirmed dysfunctional lymphatic flow in patients who have CRPS (34) or postoperative pain (38). In a report of 3 patients with CRPS and lower extremity peripheral edema who were treated with sympathectomy, lymphoscintigraphy demonstrated the affected legs of 2 patients underwent a drastic increase of lymphatic flow posttreatment. The authors proposed that sympathetic alterations caused by the patients’ CRPS increased SNS activity among the lymphatic vessels in the affected area, concluding that the ANS modulates lymphatic function (34). Among 4 patients who had postoperative pain and edema following lower extremity surgery, lymphoscintigraphy revealed increased lymphatic flow in the affected leg of all patients, with one also showing dermal backflow, a characteristic of LE (38).

Over the past decade, improved and validated techniques in lymphatic imaging have enhanced the visualization and quantification of lymphatic contractile function (39–44). Near-infrared fluorescence lymphatic imaging (NIRF-LI) visualizes lymphatic pumping in real time by tracing the flow of indocyanine green (ICG) intradermally administered at relevant anatomic sites (33, 39–44, 45–50). NIRF-LI and related ICG lymphography techniques are additionally used in clinical practice to map intraoperative sentinel lymph nodes to stage cancer, intraoperative videoangiography, and to determine breast lesion malignancy during mammography and ultrasonography (44).

We recently reported a case of CRPS with stage 1 LE following a complex leg fracture that occurred 26 years prior, whose pain at least temporarily improved following treatment with a novel pneumatic compression therapeutic (PCT) device for manual lymphatic drainage (MLD) to the patient's back that provides daily pain relief in the home setting. This Class II, PCT device is a 16-chamber mat that administers automated manual lymphatic drainage therapy (AMLDT) to the patient in a supine position (31). We observed in the CRPS and LE case that, in addition to reducing pain in the patient [based on the change in Visual Analogue Scale (VAS) scores before and after treatment], NIRF-LI captured that AMLDT additionally altered pulsatile frequency and improved lymphatic contractility, a beneficial effect that was sustained at least 30 min posttreatment (31).

The goal of this paper is to further discuss how autonomic dysfunction contributes to dermal lymphatic function and how they collectively lead to CRPS. We provide a more in-depth description of our case of CRPS and LE, as well as new evidence from this case that supports the hypothesis that stimulating dermal lymphatic contractility may improve pain management.

## 2 Lymphatic response to lower limb trauma and the potential role of MLD in pain management

Lower limb trauma has the potential to disrupt lymphatic function and lymphatic homeostasis, and can result in impaired lymphangiogenesis, limiting lymphatic recovery and reducing or significantly impairing the natural state of interstitial fluid dynamics. In an *in vivo* model of inflammation, mice were injected with lipopolysaccharide and assessed with NIRF-LI to evaluate the effect of inflammation on lymphatic function (33). Within 4 h of injury, lymphatic propulsion was inhibited, as shown by reduced lymphatic pulsing frequencies. Lymphatic vessels had noticeable dilation with increased microvascular permeability and leakage, and the same results were observed after IL-1β, TNF-α, or IL-6 was injected. Mice were pretreated with N-iminoethyl-L-lysine, which inhibited inducible nitric oxide synthase and mitigated the deleterious effect of elevated IL-1β, TNF-α, or IL-6 on lymphatic propulsion. Therefore, nitric oxide is thought to mediate the cytokine storm occurring upon inflammatory insult and in the presence of lymphatic stasis (33).

When a high-energy fracture of the lower extremity occurs, severe soft tissue disruption alters lymphatic drainage patterns, resulting in edema occurring during the acute healing phase as a normal sequela (37, 51). Persistence of edema and lymphatic dysfunction can result in loss of immunologic and soft tissue regenerative capacity if recovery is incomplete. Significantly more patients with lower extremity fractures and LE have reported pain in the affected limb compared to those without LE (64% vs. 31%, *p* = .001) (37). There appears to be a positive correlative relationship between the severity of the fracture and the development of LE. Significantly more of these patients with LE required soft tissue flaps or skin grafts (*p* = .018 and.014, respectively), had wound infections and developed osteomyelitis (*p* = .020), and developed compartment syndrome (*p* = .035). In 17 patients treated with skin grafting and muscle flaps following severe compound tibial fractures, NIRF-LI revealed that all flaps lacked functional lymphatic vessels with lymphatic blockage observed at the scar edge, and skin/muscle flaps and grafts had a dermal backflow pattern comparable to LE (51).

A PCT device is usually worn on the affected limb to increase circulation and provide MLD, a therapeutic dermal and soft tissue manipulation and massage technique that is often performed by Certified Lymphedema Therapists and that is considered standard of care to manage LE. Although lymphatic drainage patterns are highly individualized and variable, including within the same patient (31, 52), clinical evidence generated from NIRF-LI supports that MLD and PCT improve lymphatic pumping (39, 47, 49, 50). When massage-like cyclic compressive loading was administered to rats, a proliferation of immune cells to unaffected muscle was observed, demonstrating that massage induces an immunomodulatory response to inflammation, including in healthy tissue (53, 54). In 9 healthy athletes who underwent a high-intensity sprint exercise, massage inhibited the inflammatory response, as measured by reduced cytokines, including those responsible for pain, such as IL-6 and TNF-α (55). MLD was also reported to decrease edema in sprained ankles, which the authors suggested was a result of SNS inhibition coupled with induced activity of the parasympathetic nervous system (56).

Investigation into the modulating effect of MLD upon pain has produced varied results. *In vivo* research supports the beneficial effect of massage on neuropathic pain. Two weeks after undergoing spinal nerve ligation, rats had markedly elevated levels of IL-6, TNF-α, and toll-like receptor-4 (TLR4) (36). Following 2 weeks of massage, all inflammatory factors were significantly reduced. Based on these results, the authors believe that massage modulates TLR4 signaling, which in turn reduces the release of inflammatory cytokines responsible for pain (36, 57). Unfortunately, clinical trials evaluating the use of MLD on patients with CRPS have had critical design flaws and have generated conflicting results on the therapeutic benefits (58–63). Improved quantitative clinical trials and data are clearly needed to understand the potential benefit of MLD on alleviating symptoms of CRPS.

## 3 Revelations and new insight from a recent case of CRPS and LE treated with AMLDT

We recently reported that a novel AMLDT system (Neuroglide, Eva Medtec, Bloomington, MN) reduced pain and altered lymphatic contractility in 4 heterogenous cases, including 2 with chronic pain, one of which had CRPS and stage 1 LE (31). We now provide a more in-depth look at the case history of a 58-year old, white male patient with CRPS and LE, who participated in this proof-of-concept study, with additional NIRF-LI findings of the affected leg reported for the first time.

**Figure 1:**
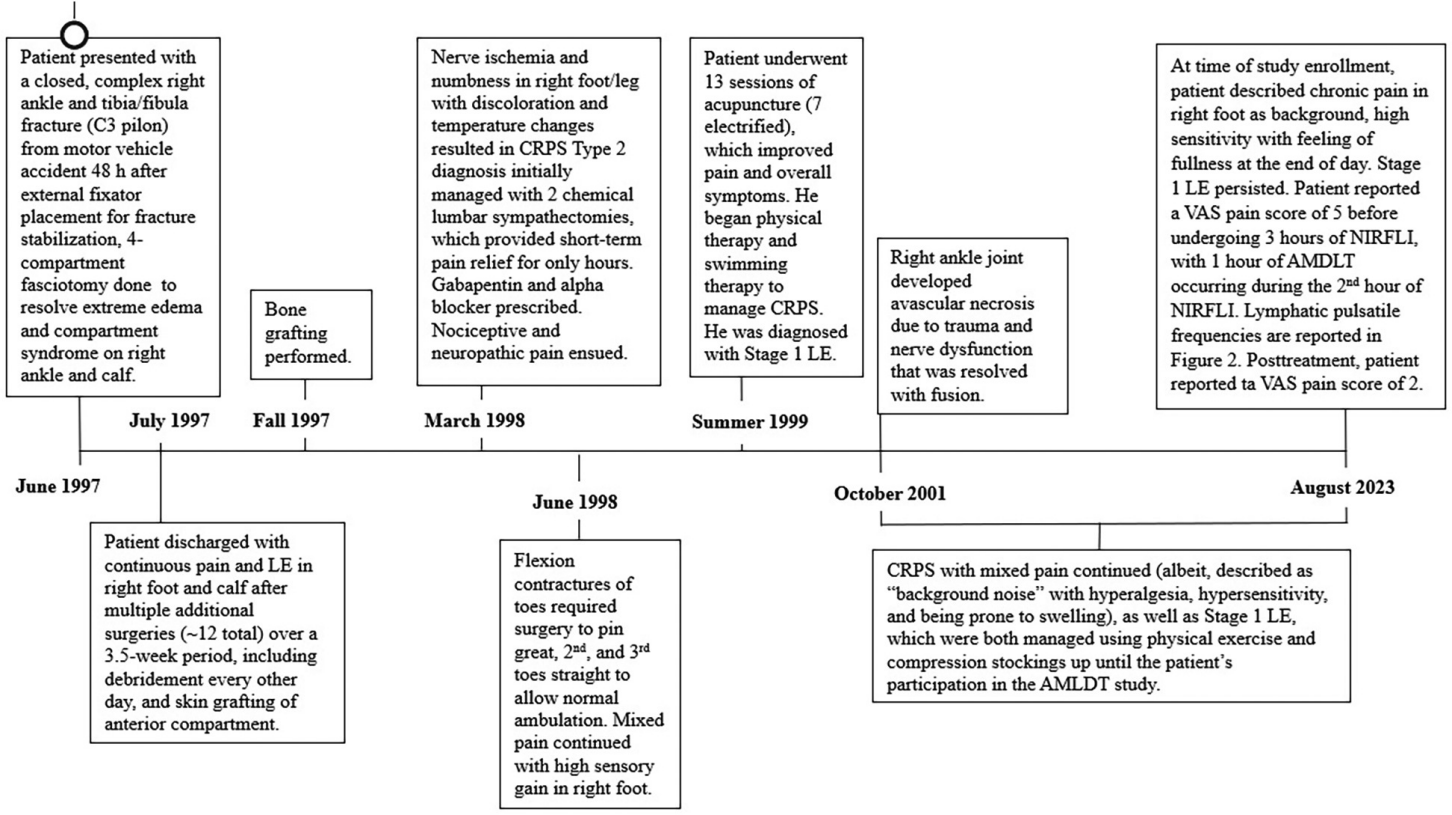
Detailed timeline of the case history of a 58-year-old male patient with type 2 complex regional pain syndrome (CRPS) and stage 1 lymphedema in the right foot and calf. The timeline covers a 26-year period of care from traumatic injury onset in June 1997 to the onset and management of type 2 complex regional pain syndrome (CRPS) in the right foot and leg beginning in March 1998 through study participation in June 2023 (31). AMDLT, automated manual lymphatic drainage therapy; NIRF-LI, near-infrared fluorescence lymphatic imaging; LE, lymphedema; VAS, visual analogue scale, based on a scale of though 10 with 0, representing no pain and 10 representing maximum pain.

Figure 1 provides a detailed history spanning 26 years from the traumatic injury onset in June 1997 to study participation in August 2023. The patient was in a high-energy, highway speed, motor vehicle accident that resulted in a complex, C3 pilon fracture of the right distal tibia, fibula, and adjacent tibial diaphyseal ankle joint fractures. He was otherwise healthy with a normal Body Mass Index and did not have other comorbidities, nor did he take medications that could have caused microvascular permeability (such as diabetes or angioedema-causing medications).

He initially underwent open reduction and internal fixation; a plate and 6 screws were inserted into the right fibula. Upon hospitalization, the patient was administered narcotics, rendering him unable to self-report his symptoms of compartment syndrome that resulted from the severity of the trauma. Consequently, compartment syndrome was diagnosed 48 h postinjury. No inflammatory markers were tested during hospitalization. Over the next 4 weeks of hospitalization, he underwent multiple extensive surgeries, including a 4 compartment fasciotomy 72 h after the injury, external fixator placement and bone fragment stabilization, multiple compartmental debridement procedures, anterior compartment split thickness skin grafting and eventually tibial bone grafting. These multiple orthopedic procedures resulted in acute microvascular hyperpermeability; consequently, extreme edema developed in the affected area of the right lower extremity (31). Upon discharge, he had continuous pain and edema and was unable to return to work for 6 months after the accident (after which he returned on part-time basis). He could not bear weight on his right foot and had to sleep with his leg elevated. After 1 year, he began to drive again, albeit requiring ongoing leg elevation, and he returned to full time work.

In March 1998, nearly 9 months after his accident, he presented to a neurologist with neuropathic pain that was secondary to nerve ischemia and was a result of the prolonged compartment syndrome in the right foot. The affected foot was noted to have bright red discoloration and vacillated between hot and cold surface temperatures. Electromyography confirmed his diagnosis of type 2 CRPS. He underwent 2 lumbar sympathectomies, which only provided short-term pain relief. [A previous case report of type 1 CRPS and edema in the leg also found that multiple lumbar sympathectomies did not provide sustained long-term relief of pain or edema (23)]. He was then prescribed gabapentin and alpha blockers to manage his mixed nociceptive-neuropathic pain; this pain medication caused a groggy state and hindered his activities of daily living. He declined opioid use. In June 1998, 1 year following the accident, the patient had impaired ambulation due to flexion contractures of the toes of his right foot. Additional corrective surgery was performed for multiple toe fusion, though the patient continued to experience mixed pain and heightened sensory gain in the right foot. CRPS sequelae were observed to be associated with the multiple, repetitive orthopedic surgeries.

A breakthrough in his CRPS management occurred in the summer of 1999, 2 years after the accident, when he underwent 13 sessions of acupuncture (including 7 sessions of electrified acupuncture), which greatly ameliorated CRPS symptoms and reduced pain. In September 1999, he declined a spinal implant during a pain consultation, and he began to manage pain with physical therapy and swimming therapy. While his pain reduced and became more manageable, his edema failed to resolve over time. He was subsequently diagnosed with Stage 1 LE, with persistent dependency swelling and skin pitting.

Four years after the accident, in October of 2001, a right ankle fusion was performed due to joint instability resulting from progressive avascular necrosis.

During the ensuing 22 years, he has managed his CRPS and LE with prescriptive gradient compression (20–30 mmHg) stockings and exercise. He more recently began taking micronized purified flavonoid fraction diosmin hesperidin (500 mg twice daily) for his LE to alter microvascular permeability and enhance vein and lymphatic tone. He reported the pain as persistent, with variable intensity (VAS daily score of 3 to 8), more akin to a “consistent background noise”. His CRPS continued to be characterized by hyperalgesia and hypersensitivity and is prone to swelling. His LE has never resolved. At the time of the study, his main CRPS complaint was that, at the end of the day, his affected foot was highly sensitive and had a feeling of heaviness and fullness.

Upon presentation at the study clinic, 32 injections of 25 μg ICG were administered to the front and back of the patient's body, including his jaw line, neck, arms, umbilical region, and feet and legs (31). One hour of NIRF-LI was performed to observe the baseline drainage of ICG laden lymph fluid. Per protocol, he next laid supine on the AMDLT mat for 1 h of treatment with concurrent NIRF-LI. A follow-up NIRF-LI assessment was done for 30 min posttreatment. He reported baseline and posttreatment pain using the VAS. Lymphatic contractility was measured by calculating pulsing frequencies before, during, and after AMLDT. NIRF-LI images were analyzed with ImageJ (US National Institutes of Health). The time stamps of the first and last NIRF-LI images determined time lapsed, and the number of pulses observed in a timeframe were counted to calculate pulsatile frequency (pulses/min).

**Figure 2:**
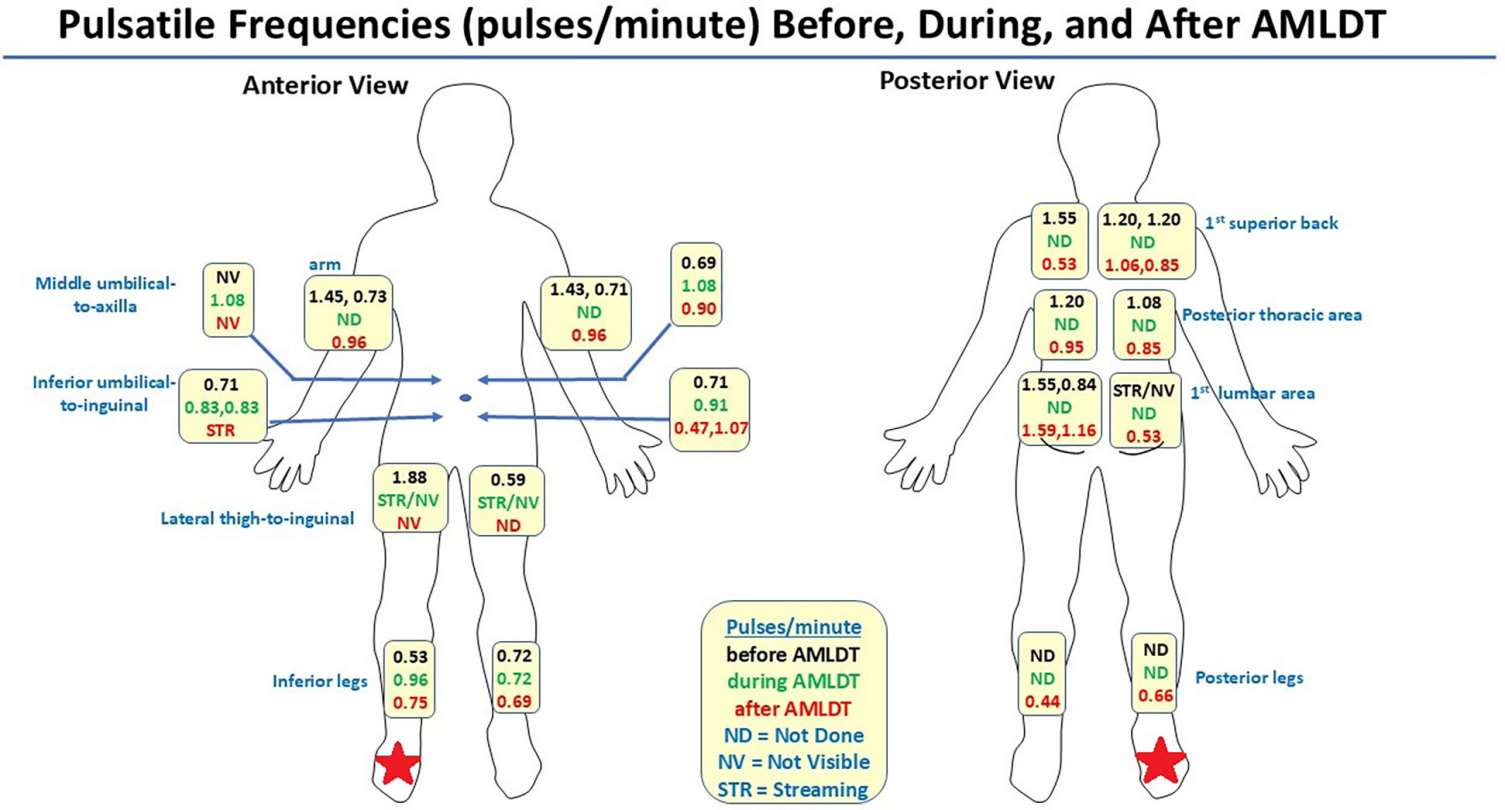
Avatar summarizing pulsatile frequencies (pulses/minute) captured by near-infrared fluorescence lymphatic imaging at anatomic indocyanine green injection sites before, during, and after automated manual lymphatic drainage therapy (AMLDT) (31). The red star indicates the limb that was affected with complex regional pain syndrome and stage 1 lymphedema.

Figure 2 depicts an avatar of the patient with CRPS and LE, showing the pulsatile frequencies obtained from NIRF-LI at the ICG-injection sites before, during, and after AMLDT. These data have already been reported and discussed in our previous publication (31), but the findings in his right foot and leg deserve further detailed notation. Lymphatic dysfunction with dermal backflow and tortuous vessels were confirmed by NIRF-LI in the calf and foot of the right leg (Figure 2 and Supplementary Video S1). The “normal” medial dorsal lymphatic collector should drain the ICG injection sites on the dorsum of the foot. Instead, drainage was routed along the lateral region of the lower right leg and into the area of dermal backflow (indicated by the arrow at the beginning of Supplementary Video S1). A medial calf incision is present from the prior 4 compartment fasciotomy that likely impairs lymphatic flow along the medial calf ventromedial bundle where no dermal lymphatic flow is identified. AMLDT increased pulsatile frequency by nearly 2-fold, from 0.53 pulses/min at baseline to 0.96 pulses/min during AMLDT (Figure 2). Supplementary Video S2 provides a sample segment during AMDLT treatment. The moving arrow indicates the movement of a bolus of ICG–laden lymph through the vessel. Importantly, posttreatment pulsatile frequencies were still elevated to 0.75 pulses/min (Figure 2) even after the AMDLT was completed. Supplementary Video S3 shows a sample posttreatment segment; the arrow near the bottom right points to the lateral lymphatic vessel on the right leg that drained into the area affected by backflow. The other visible vessels on both legs near the Achilles tendon drain the ICG injection sites near the Achilles tendon. Therefore, NIRF-LI clearly captured lymphatic dysfunction in this patient with CRPS and LE, which at least temporarily improved during and after 1 h of AMLDT.

Before treatment, the patient reported a VAS pain score of 5, which decreased by more than half to a reported value of 2 immediately posttreatment. These initial clinical data suggest that improving lymphatic contractility may reduce pain.

## 4 Discussion

The understanding that the autonomic innervation of lymphatics is critical to full functionality has been largely speculative (14). Our patient with CRPS and Stage 1 LE exhibited the dynamic, characteristic mixed pain of CRPS throughout his case history, with confirmed autonomic and lymphatic involvement (Figure 1). He had a high-energy accident with associated compartment syndrome and resulting nerve ischemia, neuropathic pain development and numbness, nociceptive pain, high sensory gain, and swelling and LE in his injured right foot and calf. We believe that his nerve dysfunction in the injured limb, which led to the development of chronic pain, is also associated with reduced lymphatic contractility and potentially some degree of microvascular hyperpermeability within the lymphatics. Collectively, the autonomic and lymphatic dysfunction contribute to the persistent negative, feedback loop of CRPS and LE.

AMDLT is indicated for pain reduction. In our reported proof-of-concept study of 4 heterogeneous cases, we demonstrated that AMLDT both impacted lymphatic function and decreased pain during and immediately following treatment (31). This noncomparative, observational pilot study was limited by a very small sample size, which limits its generalizability. A randomized controlled trial is recommended to determine the long-term effect of AMLDT on lymphatic function and pain with repeated treatments over a longer timeframe (31). Nonetheless, the additional findings reported herein from our CRPS and LE case study support that lymphatic dysfunction is associated with chronic pain, and improving lymphatic contractility in dysfunctional areas may reduce associated pain through an interplay of autonomic and lymphatic stimulation, which can be explored in future research by performing neuropathic assessment before and after repeated MLD and AMLDT treatment to validate our initial observations.

In our case report, NIRF-LI was used to visualize real-time lymphatic function; however, the quantification of lymphatic contractility is pending validation across multisite studies. In a pilot study that compared NIRF-LI on 24 healthy subjects compared to 20 with LE, lymph propagation velocity, period, and contractile frequency were significantly affected by LE status and by limb (arm or leg) (46). However, quantification was highly variable and may preclude diagnosis of LE on these measures alone. In a swine wound model, that used NIRF-LI to compare the effect of closed incisional negative pressure therapy to ipsilateral controls over 5 visits in 2 weeks, repeated quantification of propulsion rates was demonstrated (64). Lymphatic activity upstream from the incision site was observed to consistently decrease significantly from baseline over time (*p* ≤ 0.0157), while downstream rates demonstrated a significant drop immediately following surgery (*p* < 0.01), recovered to baseline by Day 6, and showed significant improvement from baseline by Day 9 (*p* < 0.0001). Automated Lymphatic Functioning Analysis (ALFIA) of NIRF-LI, an algorithm under development to automate quantitative analysis, was validated against manual analytic approaches in 9 subjects (3 healthy controls and 6 with LE) (65). ALFIA incorporates an object-tracking algorithm for visual stabilization to reduce motion artifacts, a flow map to more reliably describe fluorescence propagation, and a refinement algorithm to adjust the flow line positioning. There were no statistically significant differences in velocity and periodicity between manual analysis and ALFIA, although periodicity was slightly longer with ALFIA with more quantifiable propulsion events observed, likely due to the adjustments for subject movement and stabilization features of ALFIA (65).

Like lymphatic dysfunction and resulting LE (31, 48, 52), CRPS varies highly among and within patients over time (1, 63). We share new clinical data from a single case report with the hope that there is more dialogue on the association between CRPS and LE, which will then result in future research to generate stronger evidence. A controlled, comparative study is needed to understand the potential beneficial effect and long-term impact that improving lymphatic contractility may have on patients with CRPS, in particular, among those patients who have a history of lower limb fractures and/or postoperative sequela. Ideally, future research will involve both *in vivo* models and evaluate the impact of LE management on pain reduction, by analyzing functional interference of chronic pain over time and the impact of pain on activities of daily living to allow for a more objective pain assessment (66, 67). High-energy motor vehicle accidents and lower extremity fractures are a common event that often result in multiple surgical procedures and the development of chronic debilitating pain and chronic lower extremity edema (23, 37). Future investigations should evaluate the long-term association between lymphatic function and potential chronic pain mitigation (31, 49).

In conclusion, we report preliminary findings from a case report to build on the hypothesis that an interplay of persistent autonomic dysfunction and lymphatic dysfunction results in chronic pain, by providing clinical evidence of a case of CRPS, with mixed neuropathic and nociceptive components and persistent LE. The patient experienced pain relief and exhibited improved lymphatic contractility during and following a single 1 h session of AMLDT, which suggests that improving lymphatic contractility in LE may reduce pain in associated CRPS. Larger, longer, and controlled biochemical and NIRF-LI studies on patients with CRPS and LE are indicated to better understand the synergistic actions of the ANS and lymphatic system on the development of chronic pain and to confirm that improving lymphatic flow may alleviate and/or improve CRPS when used in a consistent manner.

## Data Availability

The raw data supporting the conclusions of this article will be made available by the authors, without undue reservation.
